# An Observational Approach to Examining White Parents’ Racial Socialization Practices With Adolescent Youth

**DOI:** 10.1111/jora.12766

**Published:** 2022-05-26

**Authors:** Chardée A. Galán, Shannon Savell, Melvin Wilson, Daniel S. Shaw

**Affiliations:** ^1^ University of Southern California; ^2^ University of Virginia; ^3^ University of Pittsburgh

**Keywords:** White, racial socialization, adolescence, color‐blind racial ideology

## Abstract

The racial socialization (RS) strategies used by White parents have received limited empirical attention. Thus, the current study examined the frequency and content of White parents’ RS messages to their White children during an observed parent–child discussion task on discrimination when youth were 14 years old. Participants were 243 White caregivers and their adolescent children (47.7% female). Overall, parents provided few RS messages, but when they did, they often relayed egalitarian messages or messages minimizing racism. Other types of RS strategies that emerged included acknowledging racism targeting people of color, discriminatory attitudes, and false beliefs in reverse racism.

Continued acts of police brutality and racially motivated hate crimes underscore the fallacy of a post‐racial America. Recent research suggests that Black adolescents aged 13–17 experience an average of five instances of racial discrimination per day (English et al., 2020), painting a bleak picture of the extent to which anti‐Blackness is woven into societal fabric. To equip their children with the skills and confidence to navigate and challenge anti‐Black racism, many Black parents use racial socialization (RS) strategies—or verbal and nonverbal messages and practices about racial issues (Lesane‐Brown, 2006; Murry et al., 2018; Umaña‐Taylor & Hill, 2020). Extant research has generally found that RS can mitigate the detrimental effects of discrimination on negative youth outcomes, highlighting RS as a culturally specific asset in Black families (Hughes et al., 2006; Wang et al., 2020).

Although emerging intervention research focused on strengthening Black parents’ RS competency is sorely needed, these efforts must be accompanied by parallel efforts to provide White youth with the skills to navigate discussions about race and racism, as well as identify and challenge racial discrimination. While past studies have disproportionately focused on the RS practices of Black parents and other parents of color, it is important to acknowledge that all parents, regardless of their race, transmit information, beliefs, and values about race and ethnicity to their children through indirect and direct processes. However, as RS is frequently assumed to be a phenomenon specific to families of color, little is known about the ways in which White parents communicate (or fail to communicate) with their children about racial topics, likely reflecting the historical privileges afforded to White people in this country (Priest et al., 2014). In fact, a recent review found that among 259 empirical articles on family RS, only 10 studies reported on the specific RS practices of White parents with White children, many of which relied exclusively on qualitative interviews or survey measures (Umaña‐Taylor & Hill, 2020). Representing a significant advance over prior research, the current study employs an observational approach to examining RS in White families, utilizing data from a videotaped parent–child discussion on discrimination when youth were 14 years old. We focus specifically on adolescencer as this represents a key period in the development of racial identity that informs how youth think about race and navigate cross‐cultural interactions (Moffitt et al., 2021; Rivas‐Drake et al., 2014). Additionally, as prior research has focused on RS in middle‐to‐upper‐class White families, an examination of how race‐related conversations unfold in White families of lower socioeconomic status (SES) is sorely needed to expand the representation of participants from lower SES in the research literature and exploratively examine RS in this understudied population. Past studies have shown that perceived scarcity of economic resources may promote racial discrimination (e.g., Krosch & Amodio, 2014; Krosch et al., 2017), suggesting that more racism may be observed in lower than higher SES families.

Although all parents transmit beliefs and values about race to their children, even if it is through silence, motivations for engaging in racial discussions are likely very different for White families compared to families of color. Many parents of color, especially Black parents, talk to their children about how to recognize and cope with racism (i.e., preparation for bias) and encourage them to take pride in their racial identity (i.e., cultural socialization; Elmore & Gaylord‐Harden, 2013). These discussions help to buffer youth of color from the deleterious effects of racism by equipping them with the confidence, knowledge, and skill to navigate racial stressors and by counteracting negative race‐related messages they may be exposed to. Egalitarian messages, in contrast, convey the importance of treating everyone equally (Bartoli et al., 2016; Hughes et al., 2006; Vittrup & Holden, 2011).

Given the historical and contemporary privileges afforded to White individuals in the United States, the content of White parents’ RS messages and their intent in communicating these messages likely differ from those of families of color. While many Black parents view “the talk” as a matter of life and death for their children, White parents, in contrast, tend to avoid talking about race with their children (i.e., White silence), likely reflecting the tendency to view “White” as the default race (Abaied & Perry, 2021; Lesane‐Brown et al., 2010; Loyd & Gaither, 2018; Rivas‐Drake et al., 2009). When White parents *do* endorse RS strategies, they most commonly report color‐blind racial messages that dismiss the significance of race (Abaied & Perry, 2021; Underhill, 2018). Color‐blind racial ideology can take various forms, such as claiming to not “see” race, denying the existence of racism by emphasizing that everyone has equal opportunities to succeed, and discouraging discussions of race (Bonilla‐Silva, 2006, 2003; Neville et al., 2013). It is also possible that some White parents may convey false beliefs in reverse racism, or messages suggesting racial discrimination against White people (Hughey, 2014). Alternatively, some White parents provide color‐conscious messages that acknowledge the reality of racism that targets people of color, celebrate racial diversity, and endorse equal treatment of all races; however, according to extant research, such messages are far less common compared to White silence and color‐blind racial messages (Abaied & Perry, 2021; Hagerman, 2014; Perry et al., 2019).

Little is known about the ways in which White parents socialize their children to think about Whiteness. According to Janet Helms, “Whiteness is a set of often unnamed and unmarked cultural and racial practices (e.g., customs, traditions), values, and attitudes that signify what is considered normative, thus privileging White skin and naturalizing systems of White supremacy.” (p. 718, Helms, 2017; Schooley et al., 2019). Helms (1990) argues that there are two broad phases of White Racial Identity Development (WRID). Phase I of WRID is characterized by racial bias, obliviousness regarding racism, and limited reflections on Whiteness and White privilege. Phase II, by contrast, is characterized by increased recognition of racism, critical introspection regarding the ways in which one benefits from White privilege, and a commitment to anti‐racist action. White silence, color‐blind racial ideology, and reverse racism messaging likely impede White youth’s progression to a more advanced WRID phase, undermining their ability to think critically about racial inequities and their own role in perpetuating racism. Alternatively, engaging White youth in discussions of racism may foster critical consciousness, or increased awareness of systems of oppression and anti‐racist action to dismantle the drivers of racial inequities (Freire, 1973).

## Current Study

The current study builds upon prior research on RS in White families by using observational methods to assess parents’ RS strategies. Research on RS has relied largely on self‐reported methods (e.g., qualitative interviews or surveys) in which parents are asked to report on the frequency with which they provide specific RS messages (Zucker & Patterson, 2018). While there are numerous benefits to self‐report, such methods are ill‐suited for capturing the subtle nuances of parents’ RS strategies, particularly implicit and nonverbal exchanges. Self‐report is also subject to social desirability (e.g., concerns of political correctness), which may be especially pronounced when asking about sensitive topics, such as best ways to respond to racial discrimination (Pahlke et al., 2012). The current study seeks to address these prior methodological limitations by utilizing an observational approach to assessing White parents’ RS strategies. In contrast to self‐report, observed parent–child interactions allow us to examine RS practices that are grounded in actual conversations between parents and youth, potentially corroborating current conceptualization of RS in White families and/or revealing new dimensions of White RS not previously represented in the literature.

Based on the important role that parents play in shaping youths’ perspectives in race, it is critical to understand how White parents view and discuss topics of racial discrimination. The primary aim of the current study was to examine the frequency and content of White parents’ RS strategies to their White adolescent youth during an observed parent–child discussion task about discrimination at age 14. We draw on a rich dataset of 243 White caregivers and youth who have been followed prospectively from child ages 2 to 14. While all discussion tasks involved primary caregivers and youth (*n* = 243), alternate/secondary caregivers who regularly cared for youth were also invited to participate in the discussion if available. Although most primary caregivers were youths’ biological mothers, approximately half of families (*n* = 116) also had a participating father figure, most of whom participated in the assessment as an alternate caregiver. We examined RS strategies assessed in prior research, such as egalitarianism, but as RS research with White families has been limited, we also explored other relevant factors, including expressing explicit racism and denying or minimizing the existence of racism.

## METHODS

### Data Source

Participants were a subsample of 243 White families drawn from the Early Steps Multisite (ESM) study (Dishion et al., 2008). The ESM project is a randomized controlled trial of 731 caregiver–child dyads who were recruited between 2002 and 2003 from Women, Infants, and Children Nutritional Supplement (WIC) programs in the metropolitan areas of Pittsburgh, Pennsylvania, and Eugene, Oregon, and within and immediately outside of Charlottesville, Virginia (Dishion et al., 2008). Families were recruited when children were between 2 years, 0 months and 2 years, 11 months based on sociodemographic risk (e.g., low family income, low parental education), family risk (e.g., parental depression, teen parent), and child risk (i.e., conduct problems, parent–child conflict). To qualify for the study, families needed to score above established clinical thresholds or be at least one standard deviation above the normative mean in two of the three risk categories.

#### Sample reduction

Based on our interest in the RS strategies used by White families, this paper focused on the 243 White caregiver–child dyads that participated in the age 14 discrimination discussion task. Families were included if the primary caregiver and youth self‐identified as White on a demographic questionnaire completed during the age 14 assessment (i.e., answered affirmatively to being White, and no to all other race/ethnicity categories).

In the analytic subsample, the average annual household income was $41,214 (*SD* = $23,343), and the distribution of families by study site was as follows: Eugene, Oregon (53.1%, *n* = 129), Pittsburgh, Pennsylvania (32.1%, *n* = 78), and Charlottesville, Virginia (14.8%, *n* = 36). Approximately half of youth were female (47.7%; *n* = 116) and half were male (52.3%; *n* = 127), and most primary caregivers were biological mothers (92.6%, *n* = 225). For a smaller subset of families, the primary caregiver was the child’s biological father (6.6%; *n* = 16) or grandfather (0.8%; *n* = 2).^1^

Of the 243 families in the current sample, 108 (44.4%) had an alternate caregiver who also participated in the age 14 assessment. The majority of these 108 alternate caregivers were father figures: 54.6% (*n* = 59) were youths’ biological father, 26.9% (*n* = 29) stepfathers, 8.3% adoptive fathers (*n* = 9), and .9% grandfathers (*n* = 1). The remaining alternate caregivers were biological mothers (3.7%; *n* = 4), stepmothers (2.8%; *n* = 3), and grandmothers (2.8%; *n* = 3).

To summarize, 236 of 243 families had a maternal caregiver who participated in the study (as a primary or alternate caregiver), 97% of whom were biological mothers (*n* = 229), 1.3% stepmothers (*n* = 3), and 1.3% grandmothers (*n* = 3). Furthermore, 116 of 243 families had a paternal caregiver who participated, 64.7% of whom were biological fathers (*n =* 75), 25% (*n =* 29) stepfathers, 7.8% (*n =* 9) adoptive fathers, and 2.6% (*n =* 3) grandfathers. As the vast majority of maternal and paternal caregivers were youths’ mothers and fathers, respectively, we henceforth use the terms “mother” and “father.” Thirty‐seven percent (*n* = 86) of mothers and 50.5% (*n* = 54) of fathers had a high school degree or less.

### Procedures

Two‐ to three‐hour assessments were conducted almost annually in families’ homes and/or laboratory setting from ages 2 to 10.5 (2002–2011), then again at age 14 (2015). As the observed measure of RS was only administered at the age 14 assessment, all data reported in this manuscript were collected at this timepoint. During study assessments, primary caregivers and target youth completed questionnaires regarding sociodemographic characteristics, contextual factors, and child behavior. Caregivers and youth were also videotaped taking part in structured and unstructured tasks that were tailored to be developmentally appropriate at each age (e.g., free play tasks in early childhood; discussion tasks during middle childhood and adolescence). Alternate caregivers—identified by primary caregivers as someone who regularly cared for the child—were also invited to participate in assessments when available, and in most cases, was the child’s father or grandmother. A subset of families in the ESM study were randomly assigned to receive the Family Check‐Up (FCU) intervention following each of the assessments from ages 2 to 10.5. See Dishion et al. (2008) for a description of the FCU. All study protocols were approved by the Institutional Review Board at each of the three study sites (i.e., University of Pittsburgh, University of Oregon, University of Virginia). Caregiver consent was obtained prior to the administration of any measures at each assessment and youth assent was obtained beginning at age 14. Families were compensated for their time.

### Measures

#### Observed measure of caregiver RS

During the age 14 assessment, primary caregivers and target youth participated in a videotaped family discussion task. Examiners provided youth with the following instructions: “Please discuss an experience of discrimination that you or someone around you has faced. Tell us how you coped, or they have coped with the experience of discrimination.” Caregivers were then asked to share the best ways to handle racial discrimination based on their cultural and family values. Families were given five minutes to discuss the topic in private while being videotaped.

To assess caregivers’ RS strategies, videotaped discussion tasks were coded using selected items from the 41‐item Observational Measure for Ethnic‐Racial Socialization (OMERS) paradigm (Yasui, 2008; Yasui et al., 2015). The OMERS coding system was developed to examine RS processes in families from diverse racial and ethnic backgrounds and was informed by focus groups, consultant input, and prior research on RS (Yasui, 2008). The initial validation study included White, American Indian, and African American adolescents and their primary caregivers, and race‐specific analyses demonstrated that all RS codes from the OMERS had internal consistencies above .80 in White families. See Yasui (2008) for more details on the development and validation of the OMERS.

The following items from the OMERS were utilized in the current study: (1) *promotion of mistrust*: messages that emphasize caution or avoidance of other racial‐ethnic groups which for White families were renamed false beliefs in reverse racism; (2) *minimization of racism:* the extent parents minimize or deny the existence of discrimination. We also coded for themes that were not included in the OMERS coding system but emerged in the current study: (1) *acknowledging racism targeting people of color:* the extent to which parents seek to increase their child’s awareness of racism against other racial groups by providing historical and current examples of discrimination; (2) *egalitarianism*: messages that emphasize individuality and treating everyone the same; and (3) *parents expressing racism and validating children*’*s racism*. Separate codes were generated for mothers and fathers. A 9‐point scale was initially used to code all variables ranging from 1 = *no evidence* to 5 = *little or some evidence* and 9 = *high evidence*. However, because of limited variability, all variables were recoded into a 3‐point scale, such that a score of 0 denoted that *parents provided no statements related to that RS strategy* (e.g., egalitarianism), a score of 1 indicated that they made *one statement*, and a score of 2 indicated that they provided *two or more statements*.

Coders were trained in the proper application of the OMERS coding manual over several training cycles in which the same sample of videos was independently coded. The lead coder then checked inter‐coder agreement for each variable, identified discrepancies, and provided written and verbal feedback to clarify disagreements on the application of codes. A coder trainee was deemed ready to code the data once they obtained satisfactory agreement (80–85%) for two consecutive videos. Coders continued to meet weekly to discuss coding questions and form a consensus as they progressed in individually rating their assigned videos. In these meetings, coders discussed general impressions from their coding and received feedback from the coding team. To minimize potential biases individual coders may have held, coders were asked to share their interpretations of statements made by participants in the videotaped family discussion tasks. Approximately 25% of the videos were randomly sampled and dual coded to assess coder reliability and ensure that inter‐rater agreement remained at 80% or higher for each coder. The overall agreement between observers was 84%. The team of trained coders included graduate students, postbaccalaureate staff, and undergraduate research assistants. Coders were from diverse racial backgrounds (e.g., Latinx, Arab, non‐Hispanic White) and predominantly self‐identified as female. During training and throughout the coding process, coders were repeatedly asked to reflect on and discuss how their social identities such as their race, ethnicity, gender, and sexual orientation may shape their interpretations of families’ interactions.

### Data Analysis

Analyses consisted primarily of descriptive statistics. To examine the frequency of parents’ RS strategies, we calculated the percentage of parents that provided each type of RS message (e.g., egalitarian messages, acknowledging racism targeting people of color), with separate scores computed for mothers and fathers. In addition to reporting on overall frequencies, we also provide quotes from actual parent–child discussions to further elucidate the nuances of White parents’ RS strategies.

## RESULTS

In the sections that follow, we report the frequencies of White parents’ RS strategies and examples of statements made by White parents and/or youth during the age 14 observational task. See Table 1 for a summary of the RS strategies that emerged in this study and the frequency with which they were discussed by families. These comments have been organized by RS strategies and are presented by descending order of frequency, specifically acknowledging racism targeting people of color, egalitarianism, minimization of racism, parents expressing racism and validating children’s racism, and false beliefs in reverse racism. To contextualize the conversations between parents and youth, it is important to note that 11% (*n* = 26) of youth described incidents in which they perceived themselves to be targets of discrimination, evincing beliefs in reverse racism; further, for 18 of those 26 families, incidents in which people of color were targets of discrimination were also discussed.

**TABLE 1:** Racial Socialization Strategies

Theme	Definition	Frequency	
		Mothers	Fathers
		*N* (%)	*N* (%)
Acknowledging racism targeting people of color	The extent to which parents seek to increase their child's awareness of racism against other racial groups by providing historical and current examples of discrimination	127 (53.8%)	57 (49.1%)
Egalitarianism	Messages that emphasize individuality and treating everyone the same	74 (31.4%)	34 (29.3%)
Minimization of racism	Messages that minimize or deny the existence of discrimination	59 (25%)	36 (31%)
Parents expressing racism	Racially discriminatory comments made by parents	47 (20%)	18 (15.5%)
Parents validating children's racism	Racially discriminatory comments made by children that were validated by parents either explicitly through supporting comments or indirectly by responding with silence	51 (21%)	34 (29.3%)
False beliefs in reverse racism	Messages that emphasize caution or avoidance of other racial‐ethnic groups and beliefs in racism that targets White people	17 (7%)	7 (6%)

### Acknowledging Racism Targeting People of Color

During the age 14 parent–child discussion task, 53.8% of mothers (*n* = 127) and 49.1% (*n* = 57) of fathers provided at least one message acknowledging racism targeting people of color. These messages came in the form of examples from television or movies, personal examples of being a bystander to racial discrimination, and historical examples. However, although several parents acknowledged racism towards people of color, the content of these messages was often very limited in detail. For example, when one girl noted that she did not understand the meaning of “discrimination,” her mother responded by stating, “Discrimination is like in that movie we watched *the Butler* about how Blacks are treated.” However, she did not elaborate any further to ensure that her daughter understood what discrimination is and quickly changed the conversation to an entirely different topic.

Nonetheless, there were a couple of parents who engaged in more meaningful discussions with their children. For example, one White mother described an incident in which her Black friend’s daughter was the target of racial discrimination. The mother explained how her friend’s daughter was prohibited from studying with one of her classmates because the other child’s parents told her that she “could not study with Black children.” The mother and her daughter agreed that this was “wrong”, and the mother went on to say, “We would never make rules like that because we have so many different cultures just in our family and that’s a silly rule and very hurtful.” The mother and child then discussed the best ways to respond when you are a bystander to discrimination or racism such as “calling it out” or “telling adults.” Another mother told her son that if he sees someone being racially discriminated against, he should “stand up and say something and not be okay with that.”

Nonetheless, the majority of White parents in this study did not provide youth with guidance on how to respond to racial discrimination. For example, one mother told her son about a recent incident in which “…a White police officer choked out a Black man.” However, she immediately changed the topic to something completely unrelated to racial discrimination, failing to elaborate on *why* Black men are disproportionately targeted by police and never providing her son with suggestions for how to resist anti‐Black racism. Overall, White parents demonstrated limited efforts to educate their children about racism and discrimination, and the messages they *did* provide often lacked detail and nuance.

### Egalitarian Statements

During the age 14 parent–child discussion task, 31.4% of mothers (*n* = 74) and 29.3% (*n* = 34) of fathers provided at least one egalitarian message emphasizing the importance of treating everyone the same. Many egalitarian statements pointed to familial values without explicitly mentioning race, such as one father who said, “As my dad taught me and as I try to teach you, be kind to others, treat them the way you want to be treated.” Another White mother told her daughter, “There is a lot of really good people that may not look like me or may not look like you, but they are great people. So, you just be nice, and you don’t judge people and you don’t put them into categories and think this or that just because and you will be better off for it.” Other parents provided egalitarian statements that directly referred to race. For example, one mother said, “Treat other people the way you want to be treated…Treat everyone the same. It doesn’t matter the color of their skin.” Similarly, one father told his son, “Treat everyone equally and don’t look down on anybody for their race or anything like that.” After a White girl told her grandmother that she had never witnessed racial discrimination, her grandmother responded by saying:

This is not something we allow as a household. I have nieces that are full blood Chinese. My daughter has expressed me this way, I am the most colorblind person she knows. I do not know color or culture. People are people. We raised them with those values that all people are created equal.

### Minimization of Racism

Twenty‐five percent of mothers (*n* = 59) and 31% of fathers (*n* = 36) made statements minimizing racial discrimination. For example, after one White boy told his parents that he had never witnessed racial discrimination, his father responded by saying, “Yeah I don’t see a whole lot of it so much anymore here.” As the father said this, the family’s dog, a Pitbull, entered the room, and the mother joked, “But Jackson gets racially profiled because he’s a Pitbull! Hahahaha!” By jokingly suggesting that their dog experiences racial discrimination, this mother significantly downplays and dismisses the impact of discrimination and insinuates that racial discrimination can be directed towards anything or anyone. Other White parents asked their child if they had ever witnessed or experienced discrimination and if they had not, the family spent the remainder of the task discussing something completely unrelated to racial discrimination. For instance, one father told his daughter: “It means you are treated differently because of the way you look or act or what you believe…Anyone at school discriminated been against because of their color?” When the adolescent girl answered “no,” her father responded by saying, “Well that’s five minutes right there,” alluding to there being nothing else to talk about for the rest of the discussion task. This father fails to appreciate that his daughter may not know how to identify something as being racially discriminatory. Rather than using this task as an opportunity to educate his daughter on these important issues, he spends the rest of the task texting and watching sports on his phone. Overall, these types of messages and behaviors socialize White youth to discount the reality of racism and to remain ignorant regarding their own role in perpetuating oppressive systems and practices.

### Parents Expressing Racism and Validating Children’s Racism

Approximately 20% of mothers (*n* = 47) and 15.5% (*n* = 18) of fathers made at least one racially discriminatory comment during the discussion task. This theme predominantly captured instances in which family members recounted past instances of making racist comments. In most instances, parents recognized that what they were saying was inappropriate and attempted to correct what they said. However, youth were often left with contradictory and confusing messages. For example, one mother described an incident in which she made racially derogatory comments towards parents at her child’s school, because she was frustrated at their inability to speak English. The mother initially attempted to justify her actions to her son by stating, “I don’t care, I did the best I could.” However, she later acknowledged, “I agree it was unfair and not right for me to feel the way I felt and say the things I did.” She ended the conversation by telling her son, “I want you to care and not act like me and to have values that I do not have because you are the next generation.” Such mixed messaging—namely, a combination of messages that justify and then condemn racial discrimination—may leave White youth confused about how to respond to witnessing discrimination in the future, particularly regarding whether they should join in or intervene, possibly allowing for the transmission of racist beliefs and actions to the next generation.

Quite disturbingly, two White families used racial slurs during the discussion task. Rather than talking about racial discrimination, one mother–daughter dyad spent the entire discussion task arguing about the child’s chores and curfew, repeatedly referring to one another as the N‐word (e.g., “Come on, N*****, stop lying!”). Another White mother turned to her husband as she said, “Like when you call me a ‘N*****.’ I don't like that!” Her husband responded by saying, “What about ‘Negro,’ is that an alright word for you?” Notably, although the boy's mother indicated that she does not like to be called the N‐word, she provided a colorblind racial explanation of why this term should not be used: “N***** is used to put people down, and I don't think highly of people who use that word!” Her husband challenged her by saying, “Well that word is not like that now,” and the conversation ended with his wife saying, “Fine, you win, I don't want to argue about this anymore.” Notably, this mother used the N‐word throughout the discussion task despite indicating that she did not approve of its use.

Several parents condoned their child's racist behavior by laughing or making comments to suggest that such racism was somehow warranted (21.0% (*n* = 51) mothers; 29.3% (*n* = 34) fathers). For example, one adolescent girl told her mother, “A girl at school told me I should date a Black guy. I said I can't date him cause he is Black. She screamed ‘RACIST!!,’ and everyone wanted to beat me up, so I punched that girl in the face and then got sent to the principal's office.” The girl's mother attempted to minimize her daughter's comments by saying, “You aren't actually racist. It was because of what your dad told you when he got to jail, that you weren't allowed to date Black guys. So, you were just repeating what your dad said.” By suggesting that her daughter was “just” repeating her father's words, this mother essentially gives her daughter an excuse to justify her racist comments. Another mother–daughter dyad had a similar discussion as the adolescent girl recalled her father telling her, “You're a slut, a whore, and a N‐word lover for dating a Black guy. You're going to end up pregnant by 14 just like your mother.” Rather than condemning the father's comments as racist, the girl's mother defended him by saying, “He was just trying to caution you because he doesn't want you to make the same mistakes we did and get pregnant at a young age.”

Other parents condoned racism in more subtle ways, often by laughing or remaining silent.

For instance, a White boy told his mother about an incident involving Black students in his class:

They were calling me cracker and I was being racist back. They kept being racist and I was being racist back because I did not like them being racist to me. They called me a cracker and I called them picker…and said go pick me some cotton.

The mother responded by laughing hysterically to her son's story. Although she eventually said, “I should not be laughing,” she made this comment as she continued laughing and struggled to gain her composure. After approximately a minute of laughing, she told her son, “You shouldn't have said those things. Well, if they say it first then I guess well (pauses) I shouldn't tell you that.” While this mother never explicitly tells her son that he was justified in making racist comments, her language implicitly suggested this.

Other parents were more unequivocal in their messaging and clearly chastised their children for making racist comments. However, of the parents who responded in this way, none of them explicitly mentioned race, racism, or Whiteness to help their children understand why their comments were inappropriate. For instance, a 14‐year‐old boy told his mother about a situation at school involving a Mexican peer: “Donald Trump wants the Mexican kid to back over the wall. So when he annoys me, we all say that we are gonna get Donald Trump on you if you don't stop. And then someone else points to him and yells, ‘Hey! Look at that burrito!” His mother responded by saying that it was “not nice to say that” because “words can hurt.” The family then switched topics to something completely unrelated, with the mother never labeling her son's comments as racist and making no effort to educate him about White privilege or encourage critical thought regarding his role in perpetuating racism. Overall, not only did some parents actively express racism, but they also validated their children's expressions of racism.

### False Beliefs in Reverse Racism

Although few parents directly told their children to be cautious of people from other racial groups, some parents warned their children of potential discrimination they may experience because they are White. Specifically, 7% of mothers (*n* = 17) and 6% of fathers (*n* = 7) provided messages that reflect beliefs in reverse racism. One mother explicitly told her daughter, “You may be discriminated against because someone doesn't like that you are White,” while another father told his son, “Discrimination would be like a group of Black people playing, and they won't let you play because you're White.” Another mother warned her daughter by saying, “You're going to have opportunities that you may get picked on because you are White.” One family discussed their difficulties with being the “only White family” living in a “mostly Black neighborhood” and feeling “left out” or “picked on.” Other White parents expressed false beliefs in reverse racism while describing their experiences navigating the welfare system: “Try to go on welfare when you're White. They don't let you. I have been discriminated for that.” or “I went to the welfare place, and I learned that they discriminate against White people. If you're blonde and White, they just expect you to have money, and that's just not okay.” These types of messages expressing false beliefs in reverse racism overlook the historical and contemporary reality of White privilege, White supremacy, and racial inequalities in the United States and function to justify continued mistreatment of people of color.

## DISCUSSION

Despite extensive research on the RS strategies used by parents of color (Hughes et al., 2006), few studies have examined the processes by which White youth learn about race and racism. The current study addressed this gap in the literature by examining the frequency and content of White parents' observed RS strategies in a sample of 243 White caregiver–youth dyads. While prior studies of White RS have relied on survey measures and qualitative interviews (Umaña‐Taylor & Hill, 2020), the current investigation is among the first to utilize an observational approach to assess White parents' RS strategies. Consistent with the small body of research on White RS (Pahlke et al., 2012; Vittrup, 2016; Zucker & Patterson, 2018), findings indicated that when given the opportunity to educate their children about racial discrimination during an observed parent–child discussion task, most White parents had little to say. Research suggests that White parents' avoidance of racial discussions with their children often stems from their own discomfort with navigating these conversations (Hagerman, 2014; Hagerman, 2017; Hamm, 2001) and from an unfounded fear that talking about race will make their child racist (Vittrup, 2016). When White parents in our study *did* engage in RS, they often relayed color‐blind racial messages or minimized racism as a “non‐issue.” Although such messages may be well‐intended, they minimize White privilege and racial inequities and communicate to youth that they should not notice race (i.e., color‐blind RS) or talk about race (i.e., color‐mute socialization) (Abaied & Perry, 2021; Sullivan et al., 2021; Vittrup, 2016). It is unlikely that White youth who receive such messages will develop the knowledge, skills, and motivation to identify and challenge racism and racial inequalities.

While a substantial percentage of mothers and fathers provided historical or contemporary examples of racism (i.e., acknowledging racism targeting people of color), the scope of these conversations was quite limited, often comprising a single sentence or two about the realities of racism in America. Furthermore, parents often provided conflicting messages when engaging in RS. Similarly, a study of RS strategies in affluent White fathers found that despite identifying as “progressive” parents committed to raising anti‐racist children, White fathers “both challenged and reinforced hegemonic whiteness” (Hagerman, 2017). For example, the author describes a father who expressed pride in coaching a racially diverse soccer team and providing opportunities for his sons to form interracial friendships. However, the same father also reinforced negative stereotypes about Black fathers during a qualitative interview with the investigator, commenting on the absence of Black fathers at games and practices (Hagerman, 2017).

Taken in sum, the conflicting messages provided during RS and superficial explanations of racial discrimination provided by White parents underscore the limitations of focusing on the frequency of parents' RS strategies. For example, it is difficult to imagine that youth whose parents provided a one‐sentence explanation of how people of color have historically been discriminated against will have the knowledge, skills, and motivation to identify and challenge racism and racial inequalities. As posited by Anderson and Stevenson (2019) in the Racial Encounter Coping Appraisal and Socialization Theory (RECAST) model, the sheer quantity of parents' RS messages may be less important than their quality. The RECAST model emphasizes that parents' RS competency is critical to ensuring that youth not only receive RS messages but also understand and are able to use them to navigate in‐the‐moment racial encounters. Although this model has predominantly been discussed in relation to how Black parents prepare Black youth to cope with racially stressful encounters, future research examining the model's relevance to other racial groups, including White families, would be an immense contribution to the field.

It is also important to note that data for the present study were collected between 2015 and 2017, a period characterized by increases in racist political rhetoric and racial hate crimes. Future research is needed to examine the extent to which White parents have modified their RS strategies based on the highly publicized acts of racial violence and reemergence of the Black Lives Matter movement in 2020, which have brought renewed urgency to combating racism and White supremacy. Indeed, Abaied et al. (2021) found that discussions of race‐related current events were twice as common in White families in 2020 compared to 2019, which the authors attribute to race‐related events, including police violence against Black Americans and the resulting Black Lives Matter protests. However, while more White parents talked to their adolescent children about racial topics in 2020 than 2019, the quality of RS strategies worsened, as fewer parents provided color‐conscious messages (Abaied et al., 2021). These findings further underscore the need for a competency perspective to RS (Anderson & Stevenson, 2019) and highlight the importance of understanding White parents' RS strategies within the current sociocultural and political context.

We were hoping to examine the ways in which White parents socialize their children to think about Whiteness (e.g., by referencing White Supremacy or acknowledging White privilege). However, we were unable to incorporate discussions of Whiteness into our thematic analysis as such conversations were virtually nonexistent in our sample. The only exceptions were parents who expressed false beliefs in reverse racism by warning their children that they may be discriminated against for being White or by sharing instances in which they perceived themselves to be the victim of racial discrimination. Of the parents who acknowledged racism targeting people of color, none incorporated Whiteness into their explanations. These parents described racial discrimination as something that is experienced by people of color, but never acknowledged White people as inflictors of such mistreatment. Not mentioning Whiteness in racism‐related discussions allows White people to avoid confronting their own privilege and potential complicity in racism, as well any discomfort that may accompany such realizations. If we hope to make meaningful progress toward racial equality, then we must begin to name Whiteness in our conversations with White youth and encourage them to think more critically about the privileges attached to their own Whiteness. It is also important to note that White parents' failure to name Whiteness in their discussions with youth may suggest that these parents themselves may be in Helms's (1990) Phase I of WRID and are thus not equipped with the knowledge and skills to help their children progress to a more advanced WRID phase. Thus, fostering critical consciousness and a commitment to anti‐racist action in the next generation of White youth likely requires cultivating these skills in White parents as well.

It is concerning, but perhaps not surprising, that several White parents minimized the reality of racism for people of color, expressed false beliefs in reverse racism, made racist comments, and/or condoned their child's racism. Although a growing body of empirical and theoretical research has documented the intergenerational effects of racism on families of color, there has not been enough scholarly work on the intergenerational transmission of racism within White families. We posit that White parents' RS—specifically, racist comments and messages that minimize the reality of racism or express false beliefs in reverse racism—is a key pathway underlying the transmission of racism across generations. Notably, past studies of RS in White families have generally not examined false beliefs in reverse racism or explicit racism as RS strategies, which may reflect the disproportionate use of self‐reported methods in past work (Vittrup, 2016; Zucker & Patterson, 2018). An observational approach to assessing White parents' RS strategies may have allowed us to better capture the nuances of parent–child discussions concerning racism, including the less socially desirable aspects of these conversations such as explicit racism. Although less likely, differences in findings may also be due to the current study's focus on adolescence compared to prior studies which have focused on early and middle childhood (e.g., Vittrup, 2016; Zucker & Patterson, 2018). It is possible that parents of adolescents may feel more comfortable sharing overtly racist beliefs with their children compared to parents of younger children.

### Limitations and Future Directions

First, for the observational discussion task at age 14, families were broadly instructed to talk about discrimination and best ways to respond to these experiences based on their family and cultural values. An advantage of this wording is that it allowed us to observe how White parents talk to their children about racial discrimination when they are provided with few guidelines on how to structure their conversation. Nonetheless, instructing families to talk about more specific race‐related issues such as police brutality may have improved our ability to capture a broader array of RS strategies. This approach may have also yielded more RS about Whiteness, such as White identity or White privilege, which were virtually nonexistent in the current study; the only exception were parents who warned their children that they may be discriminated against for being White (i.e., false beliefs in reverse racism). Alternatively, providing families with more specific discussion topics may artificially encourage conversations that would not otherwise occur organically and prompt family members to incorporate language they would not typically use. Indeed, White parents were more likely to mention “Whiteness” or “White supremacy” when they were asked about “Current events related to White supremacism in America” compared to parents who received prompts that did not include “White” in the wording (Abaied et al., 2021). Thus, how race‐related prompts are worded seems to have important implications for the content and frequency of RS messages that are observed by researchers and should be carefully considered when designing future studies of RS in White families.

Furthermore, data for this report come from a larger study that was not specifically designed to answer questions about RS. Incorporating survey measures of parent RS and measures of parents' and youths' racial attitudes and White racial identity would allow for a more nuanced understanding of the ways in which White parents socialize their children to think about race and elucidate potential discrepancies across methodology (i.e., observational versus questionnaire) and informants (i.e., parent versus youth).

As prior research has predominantly focused on the RS strategies of mothers (Bonilla‐Silva, 2006; Bonilla‐Silva, 2003; Hagerman, 2017; Pahlke et al., 2012; Perry et al., 2019), the ability to also examine the RS strategies in a relatively large subset of a White fathers represents an important contribution of the current study. Unfortunately, our coding system did not allow us to compare the RS strategies of mothers and fathers. Although there is some research comparing the RS strategies of Black mothers and Black fathers (Brown et al., 2010; McHale et al., 2006), RS differences based on parent gender have not been explored in the small body of research on RS in White families. This represents an important area for further investigation.

It was worth noting that the current sample, albeit including parents from urban, suburban, and rural communities, was of low SES. However, as most research on White RS has been conducted in middle‐ to upper‐class families (Hagerman, 2017; Underhill, 2018; Vittrup, 2016), our use of a low‐income sample represents an important contribution to the literature. In fact, Underhill (2018) acknowledges that her findings are limited in generalizability to middle‐class families and notes, “It is possible that poor whites are less capable of cultivating a childhood structured according to racial silence due to resource constraints. Additional research is needed to assess whether white racial silence is a protective practice all whites engage in, or whether it is specific to whites with class privileges” (p. 1949). Findings from the current study provide preliminary evidence to suggest that racial silence is not specific to higher SES families but is also highly prevalent among White families living in poverty. However, it is nonetheless possible that the factors motivating White parents' silence regarding racial matters may differ by class. For example, while Underhill (2018) found that many White parents expressed a desire to protect their children from “unhappy racial conversations,” such as news of racial violence or police brutality, it is unclear if racial silence is similarly used as a protective parenting strategy in lower SES White families. As our sample was comprised entirely of lower SES families at the time of recruitment, future research would benefit from incorporating more diverse samples with respect to SES to allow for a more direct and nuanced comparison of RS in higher versus lower SES White families.

## CONCLUSION

The dual‐pandemics of 2020–2021—the COVID‐19 and racism pandemics—have laid bare deep‐seated disparities in education, healthcare, and safety between White people and racial and ethnic minorities. If we strive for a country that is free of racial inequality in the coming decades, we must consider how shaping that future for our youth begins today. Findings from the present study highlight the many ways in which White parents are falling short in preparing White youth to recognize and challenge racial discrimination targeting youth of color. For White parents, this means abandoning silence and colorblind racial ideology as primary modes of RS and instead, proactively engaging their children in discussions of racism and equipping them with the skills to challenge White supremacy (Galán et al., 2022). Although it is easy to avoid discussions of race when they feel irrelevant to your child's day‐to‐day existence, parents of color do not have the privilege of deciding whether to have these discussions with their children. Until White parents begin to have these difficult but necessary conversations with their children, the burden will continue to fall on parents of color to prepare their children for experiences of racial discrimination.

## DECLARATIONS OF INTEREST

None.

## POSITIONALITY STATEMENT

The first author self‐identifies as a first‐generation college student and Mexican American daughter of immigrants; the second author self‐identifies as a White woman; the third author self‐identifies as an African American male; and the fourth author self‐identifies as a White male.

## Footnotes

^1^
Youth were provided with the following options for self‐reporting gender identity: “male”, “female”, and “transgender.” None of the adolescents in this sample identified as transgender. Nonbinary gender identity was not included as a potential response option for youth. There was also not an option for parents to select transgender or to express nonbinary gender identity. These are important limitations in the data collection process, and future research on racial socialization in White families would benefit from more comprehensive and inclusive practices for collecting data on gender identity.
